# Rötheln: A Summary

**Published:** 1893-09

**Authors:** John Meredith


### RÖTHELN: A SUMMARY.

BY

JOHN MEREDITH, M.D. Ed.

IN the *Bristol Medico-Chirurgical Journal* for March, 1892, there is, to me, an exceptionally interesting article by Dr. Fyffe on an outbreak of Rötheln. The district of Wellington in Somerset, experienced an extensive outbreak of this exanthem during the year 1892. I took notes[1] of a great number of the cases

[1] Some of these were published in the *Lancet*, July 22, 1893.

that came under my notice. There were twenty-five cases at our County School, among about sixty boarders: in three of these there was profuse desquamation, the cuticle separating in flakes and strips; one of them was an instance of what Dr. Fyffe describes as the recrudescent form. The others had only slight peeling, while all were similarly situated. Cases existed in the town long before the school was invaded.

The illness, while it chiefly attacked the young, did not spare the old. Persons of all ages experienced it: two, who were well over sixty, had it in unmistakable form; one of them had much desquamation, the other hardly any.

As to the character of the rash, my experience in regard to it is similar to that of Dr. Fyffe, with this exception: Dr. Fyffe describes the rash in some of his cases as being uniformly red over the body; I have not been able to fully verify this, either at the school or among the cases attended at their homes in the town. In many the rash might appear uniform as a blush when looked at from a distance, or in a defective light; but on close examination in good daylight the eruption was found to be divided by numerous narrow meandering streaks of clear skin. In a few cases I observed that the rash on the arms, legs, or nates was uniform, and no pale interspaces could be detected, but over the trunk in such cases it was spotty. There is no reason that I know of why these conditions might not be reversed. I can fully corroborate Dr. Fyffe's remarks about the rash presenting difference of character in different cases. It was possibly after observing this Dr. Brown suggested that the exanthem had "two varieties of rash—one scarlatinal, the other rubeoloid;" and he might have added varicelloid for a third, but as a rarer form.

As an example of this third, I may mention that of H. B., aged 2, whose mother had an attack of rubeoloid rötheln of a mild character, and peeled freely; while the child had only clusters of pimples at different parts of the body, and no other form of eruption. He had induration of the post-cervical glands. His play-fellows had rötheln as well as his mother. Two other little patients whom I attended had similar eruptions. These papules or pimples had white centres looking like pus, but they did not break and nothing escaped from them. As these cases were associated with ones suffering from marked rötheln, I felt constrained to look upon this chicken-pox form as only a variant of rötheln.

I found several of the cases which I attended in the town had diarrhœa and sometimes vomiting, as well as the rash, and other characteristics of rötheln existing at the same time. In nearly all there was tickling, itching of the skin, spinal tenderness was noticed, and often one of the first symptoms observed in children was seeing them placing their hands on the nape of the neck. In a few the temperature rose to 103° or 104°, with some delirium; pretty free epistaxis occurred occasionally as a consequence of vascular tension. Otitis, followed by abscess and purulent discharge from the ear, happened in several, and in a few suppuration took place behind the ear as well. I did not personally see an instance of optic neuritis, which would be a natural associate of suppuration of the middle ear in this disease, seeing that there is direct lymphatic communication between the sites. There have been several cases where the glands below the angle of the jaws became enlarged on both sides. I noticed again and again that these swellings were found to be most marked in unhealthy dwellings and among uncleanly people. I know no form of illness that is more influenced by insanitary surroundings than rötheln.

The illness as a rule is not accompanied by a sense of prostration, such as one is accustomed to see in influenza; but a patient suffering from rötheln, if confined indoors for long, acquires a peculiar pallid appearance, and is irritable rather than prostrate. As for those whom I attended I kept them in bed, or tried to, during the continuance of the febrile state, which in the great majority of cases was slight; then let them get up, and out too if the weather was fine, and not in a single case was there any unfavourable result. Those that were most out in the open air and under healthy surroundings got on best.

Desquamation is an irregular feature in this ailment; as a rule it is fine, or what is termed "branny;" but cases are plentiful where the peeling is profuse, the cuticle separating in strips and flakes from hands and feet, as well as from the trunk.

In a few, abscesses formed in the tonsils, while several complained of pain on swallowing during the first two or three days of the illness. This might be mistaken for a diphtherial feature, and I think it has been sometimes.

The relapsing or recrudescent feature is peculiar. Two young men, unconnected with one another, experienced it after an interval of fourteen days' apparent freedom : the rash came out when they felt cold, and disappeared as soon as they got warm ; each of them noticed this several times. The interval in others was shorter. The eruption in the recurrent form appeared mottled.

The exanthem, as stated, attacked a great number of persons in this district during 1892 ; it is impossible to give even an approximate idea of the number, as many did not come under the notice of medical practitioners at all. There have been some fatal cases, seven in all, in which, whilst rötheln was the primary, catarrhal pneumonia, suppurative otitis, or great enlargement of the glands in the parotid regions was the secondary cause. In no case was I able to find albumen in the urine. The period of incubation seems to me to be uncertain ; while a fortnight appeared to be the time in some cases, in others it was longer.

Like any other exanthematous ailment, it is not easy at the commencement to give a definite diagnostic sign of rötheln ; but when one meets with a case where the eyes are suffused, the sub-occipital or post cervical glands thickened, and the body in some places covered with a reddish or pinkish rash — the eruption consisting of minute elevated spots—the presumption is that the case is one of rötheln. I will conclude my remarks by quoting a statement of Dr. Tonge-Smith regarding the diagnosis of this disorder. " There is seldom," he says, " any difficulty if the case be seen by daylight and all the signs be taken together, with a mind not[1] bent on discovering the wonderful and ignoring the simple."

[1] *Lancet*, 1883, vol. i., p. 1036.

